# IMPROVING AGING IN PLACE FOR OLDER VETERANS LIVING IN PERMANENT SUPPORTIVE HOUSING

**DOI:** 10.1093/geroni/igad104.3127

**Published:** 2023-12-21

**Authors:** Kelly Su, Medha Romee Maitra, Amanda Peeples, Octavia Goodman, Rebecca Brown

**Affiliations:** Cornell University, Ithaca, New York, United States; University of Georgia, Athens, Georgia, United States; Corporal Michael J. Crescenz VA Medical Center, Philadelphia, Pennsylvania, United States; Corporal Michael J. Crescenz VA Medical Center, Philadelphia, Pennsylvania, United States; University of Pennsylvania, Philadelphia, Pennsylvania, United States

## Abstract

The average age of formerly-homeless Veterans living in HUD-VASH (Housing and Urban Development-Veterans Affairs Supported Housing) aged 50 and older is increasing. Homeless-experienced people experience accelerated aging, with premature onset of geriatric conditions and mortality. However, current supportive services at HUD-VASH do not address these conditions and enhance “aging in place,” i.e., the ability to live comfortably and safely in one’s own home and community. We conducted qualitative interviews and focus groups to determine barriers, facilitators, and adaptations needed to successfully implement interventions in HUD-VASH. We then used a modified Delphi process to reduce a list of 66 intervention elements based on perceived feasibility and importance. In total, 9 Veteran Affairs HUD-VASH staff members and 1 Veteran participated in 3 conference calls and surveys. The top-rated intervention elements were organized by: who should be involved in delivering the intervention (social workers, physicians, nurse practitioners, physician assistants, nurses), what types of goals/needs they will focus on (geriatrics, mental health needs, general health goals, dementia care, medication management), where and how should the intervention be delivered (in the home, at the VA), how long should the intervention be (when veterans are experiencing functional impairment, memory impairment, mental health problems, trouble caring for themselves, and when they are new to HUD-VASH) and when should the intervention be delivered (regularly scheduled and ongoing as needed). With feedback from expert stakeholders, our findings will be used to develop and implement interventions to improve aging in place for older Veterans living in HUD-VASH.

